# Acute Hemodynamic, Metabolic, and Hormonal Responses to a Boxing Exergame with and without Blood Flow Restriction in Non-Athlete Young Individuals

**DOI:** 10.3390/sports12030068

**Published:** 2024-02-23

**Authors:** Zohreh Karimi, Zeynabalsadat Mousavi, Michael Nordvall, Alexei Wong, Reza Bagheri, Frederic Dutheil

**Affiliations:** 1 Department of Physical Education and Sport Sciences, Science and Research Branch, Islamic Azad University, Tehran 1477893855, Iran; zohreh.karimi1@gmail.com; 2 Nutrition and Food Service, Imam Khomeini Hospital Complex, Tehran University of Medical Sciences, Tehran 1416634793, Iran; 3 Department of Health and Human Performance, Marymount University, Arlington, VA 22207, USA; mnordval@marymount.edu (M.N.); awong@marymount.edu (A.W.); 4 Department of Exercise Physiology, University of Isfahan, Isfahan 8174673441, Iran; will.fivb@yahoo.com; 5 Physiological and Psychosocial Stress, CHU Clermont-Ferrand, University Hospital of Clermont-Ferrand, Preventive and Occupational Medicine, Witty Fit, Université Clermont Auvergne, CNRS, LaPSCo, F-63000 Clermont-Ferrand, France; fred_dutheil@yahoo.fr

**Keywords:** boxing video game, blood pressure, lactate, growth hormone, heart rate variability

## Abstract

Background: This study aimed to compare acute hemodynamic, metabolic (glucose and blood lactate concentrations), hormonal (growth hormone and normetanephrine), heart rate variability (HRV), and rating of perceived exertion (RPE) responses before and after bouts of a boxing exergame with and without blood flow restriction (BFR) in non-athlete young individuals. Methods: Fourteen participants (age: 30 ± 10 y; BMI: 21 ± 3 kg.m^−2^) participated in two sessions of a 20 min boxing exergame. During week one, the participants were randomly divided into two groups and played against one another under normal (n = 7) and BFR (n = 7) conditions. Over the next exercise session, participants were then reallocated to the opposite condition (normal vs. BFR) for data collection. Hemodynamic, metabolic, HRV, and hormonal parameters were measured before and immediately after the exercise protocols. Results: Playing exergame led to a significant increase in hemodynamic variables (except for diastolic blood pressure) regardless of BFR condition with no between-group differences. Regarding HRV, significant reductions in total power (TP) and low-frequency (LF) waves were identified in the non-BFR group (*p* < 0.0001) compared with the BFR group. Conversely, a significant increase in very LF (VLF) waves was noted for the BFR group (*p* = 0.050), compared with the non-BFR group. Significant increases were observed in serum concentrations of growth hormone, normetanephrine, and blood lactate concentration from pre- to post-exercise under both conditions (*p* ≤ 0.05), with no significant differences between the groups. Moreover, no statistically significant changes were observed in glucose levels. RPE responses were significantly greater (*p* ≤ 0.05) in the BFR group compared with the non-BFR group throughout the exercise session. Conclusions: We observed similar hemodynamic, hormonal, and metabolic responses after an acute boxing exergame session in young individuals, whether conducted with or without BFR. However, notable differences were observed in certain HRV markers and RPE. Specifically, the inclusion of BFR resulted in an elevation of VLF and a heightened perceived exertion. These findings suggest that BFR may alter cardiac autonomic and perceptual responses during exergaming. Further research is warranted to understand the long-term implications and potential benefits of incorporating BFR into exergaming routines.

## 1. Introduction

A sedentary lifestyle, or sedentarism, is recognized as a global public health concern that leads to numerous morbidities [1]. While engaging in media such as video games is traditionally associated with a sedentary lifestyle, combining video games with movement encourages physical activity and may help achieve exercise recommendations [2] while being an enjoyable form of home exercise [3]. Active video game systems, such as Nintendo Wii and Kinect for Xbox, have recently become popularized, leading to the coining of the term “exergaming” in research and development as a type of entertainment that combines physical activity with video gaming [4]. Studies have shown that exergames can promote moderate to intense levels of physical activity and be used as an alternative form of exercise in the general population [5]. Enhancements to traditional exercise training, such as incorporating blood flow restriction (BFR), have been proposed to significantly improve aerobic conditioning and muscle strength without the need for high-intensity training [6]. Research has indicated that a combination of aerobic exercise with BFR results in improved acute and chronic neuromuscular and metabolic responses to exercise and more significantly increases hemodynamic outcomes compared with exercise alone [7]. This modality permits the attainment of physiological adaptations traditionally associated with high-intensity training regimes, albeit at markedly lower exercise intensities [8]. Specifically, BFR induces a spectrum of physiological effects, including augmented muscle protein synthesis, facilitated by an elevated anabolic hormonal environment; enhanced muscle hypertrophy and strength gains through metabolic stress and muscle fiber recruitment patterns that mimic those observed in high-load resistance training; and improvements in vascular function due to increased shear stress [9]. Furthermore, BFR has been shown to elevate lactate concentration and promote systemic hypoxia, which are critical factors in stimulating aerobic and anaerobic metabolism, thereby improving endurance and muscular efficiency [10]. In essence, BFR offers a mechanism by which training intensity can be intensified without the corresponding increase in load, thereby holding significant promise for advancing sports performance by amplifying the physiological impacts of training sessions [8,10]. Bridging the gap between these innovative training methods and their physiological impacts, the convergence of technology and physical exercise opens a new chapter in understanding and optimizing the human body’s response to exercise.

This intersection of physical activity and technology has paved the way for innovative approaches to exercise monitoring and effectiveness assessment. It is well established that increased heart rate (HR) and myocardial oxygen consumption during exercise result from increased sympathetic stimulation [11,12]. Recent advances in human–computer interaction, specifically in the field of physiological telemetry, have allowed heart rate variability (HRV) analysis to be a cornerstone of monitoring the effectiveness of physical exercise through exergame [13]. Using short-term Fourier transform time–frequency analysis, recent investigations have shown that HRV decreases following moderate to high-intensity exercise [14]. In addition, recent data suggest that peak lactate accumulation is higher immediately post-exercise under BFR conditions [15]. Considering the anabolic effects of lactate, it is possible to improve muscle hypertrophy using BFR compared with exercise alone [16]. Moreover, a session of aerobic exercise significantly reduces blood sugar levels (glucose) compared with resistance exercise in women with type 2 diabetes [17] and provides a stimulus for the release of growth hormone (GH) [18]. Physical stressors, such as sport-related activities, stimulate the release of normetanephrine at all workloads potentially indicating a method of determining exercise intensity and exercise performance [19]. In contrast, the release of epinephrine increases during workloads greater than 61% of VO_2max_, which, in part, may be responsible for the increased HR and blood pressure (BP) responses [20]. Therefore, any increase in plasma levels of normetanephrine, a metabolite of norepinephrine, indicates increased activity of the sympathoadrenal system [21]. Metrics such as HRV, BP, lactate accumulation, and assessments of blood glucose, GH, and normetanephrine levels, offer insights into the acute neuromuscular, metabolic, and hemodynamic responses to exercise, facilitating a comprehensive understanding of bodily adaptations to physical stressors. Given these insights into the physiological responses to various forms of exercise, the next logical step is to leverage these advancements in technology and our understanding of exercise physiology to develop engaging and effective exercise interventions, particularly for populations at risk of sedentarism.

Due to the increasing popularity of computer games among youth and the amount of time spent playing these games, alternative strategies to address sedentarism and associated morbidities are warranted, particularly in these populations. Integrating innovative exercise approaches that are both enjoyable and offer additional benefits, such as increased physical activity and improved fitness, represents a logical solution. This is particularly relevant when these approaches are combined with other stressors, such as BFR, to enhance the intensity of the exercise stimulus. By increasing the intensity, the physiological responses are subsequently heightened, thereby leading to amplified positive effects. Thus, the objective of this investigation was to evaluate the acute responses in hemodynamic, metabolic, HRV, and hormonal parameters following a boxing exergame session with and without BFR in non-athlete young individuals. The underlying hypothesis examined in this study was whether the inclusion of BFR provides additional changes in terms of hemodynamic, hormonal, and metabolic responses during exergaming among this population.

## 2. Methods

### 2.1. Participants

In the present study, 14 healthy young individuals (female [n = 8], male [n = 6]; age = 29.9 ± 7.04 y; BMI = 22.4 ± 3.46 kg.m^−2^) participated in a boxing exergame activity under normal and BFR conditions. The inclusion of both male and female participants was predicated on the understanding that exergame audiences encompass both genders and thus our research design intentionally sought to mirror this diversity to ensure the generalizability of our findings. The evaluation of participants’ health status encompassed a thorough assessment of their general physiological state and wellness. This assessment included an analysis of their medical history and an examination for the presence of known diseases. Additionally, their consumption of alcohol and drugs, dietary habits, and levels of daily physical activity were meticulously scrutinized. The physical Activity Readiness Questionnaire (PAR-Q) [22] and medical health questionnaire were used to collect the required data.

The participants were considered non-athletes (no recent participation in organized sport), had not partaken in a regular exercise program within the past year, and were not limited in their ability to participate in the boxing exergame exercise protocol. Following an explanation of the research protocol, participants were asked to complete an informed consent form in the presence of a witness to participate in the study. Participants were instructed to maintain regular sleep patterns and activities of daily life and avoid strenuous physical activity, dietary supplements, medication, cocoa, coffee, caffeinated beverages, alcohol, and tobacco for up to 48 h prior to boxing exergame sessions and data collection. In addition, participants were instructed to maintain their usual dietary habits, which included a light dinner the night before the exercise test. This was verified by reviewing their 24 h dietary recall questionnaires. It was confirmed that the caloric intake of all participants on the day prior to the test matched their required intake for weight maintenance, ranging from 1600 to 2200 kcal. They were also provided a breakfast of similar caloric value at the testing site, in a controlled environment. This breakfast included two slices of toast, a slice of sausage, and Gouda cheese, amounting to approximately 32 g of carbohydrates, 10 g of protein, 12 g of fat, and a total of 300 kcal. This research received the approval of the Research Ethics Committee of the Islamic Azad University of Tehran, Iran, Science and Research Branch, with the ethics code of IR.IAU.SRB.REC.1399.100 and IR.IAU.SRB.REC.1399.101 and was conducted in strict adherence to the principles outlined in the Declaration of Helsinki.

### 2.2. Study Design and Exercise Protocol

This investigation utilized a crossover design encompassing 14 participants who were randomly assigned to two groups: one subjected to a boxing exergame with blood flow restriction (BFR), and the other to an identical exergame without BFR. The study commenced with a session of both groups partaking in the boxing exergame. This was followed by a one-week washout phase, after which the groups switched conditions for the second week, thereby replicating the exercise regimen. Preceding the study’s initiation, participants were bifurcated into gender-specific cohorts, within which they were further randomized and paired. In preparation for the study, a familiarization phase was conducted, during which participants familiarized themselves with the study environment, the boxing exergame, and the research methodologies. Baseline anthropometric measurements, including height and body mass, were ascertained a week prior to the protocol commencement, utilizing a Seca 206 wall-mounted stadiometer (SECA, Hamburg, Germany) and a BF800 Beurer digital weight scale (Beurer, Söflinger Straße, Germany), respectively.

Participants were instructed to report to the exercise laboratory at 7:00 am for the boxing exergame sessions. All assessments were conducted in the morning (8:00–11:00 am) under controlled ambient laboratory conditions (temperature: 24 °C; humidity: 42%). Each session was initiated with a 5 min warm-up comprising stretching exercises. Resting blood pressure (BP) was evaluated in the supine position in the morning prior to exercise testing, using Microlife BP A100, a digital sphygmomanometer (Microlife, Düsseldorf, Germany), with measurements taken thrice and averaged. Relative arm occlusion levels for each participant were set at 15–20% below their systolic BP (SBP).

The selected exergame was boxing from XBOX360 Kinect Sports (Microsoft Game Studios, Redmond, WA, USA). Participants were randomly divided into two groups, and each group engaged in a 20 min session of an exergame that involved hand punches and movements resembling those used in boxing. In the initial session, seven participants with BFR (induced via an Iranian-made inflatable cuff set to 15–20% SBP restriction [23]; the cuff width employed was 6 cm) competed against seven participants without BFR. The BFR was applied by encircling the upper arm near the shoulder with the cuff. After a seven-day interval, the groups reconvened, swapping conditions, and the exergame session was repeated. BFR conditions involved intermittent cuff inflation and deflation at 5, 10, and 15 s intervals. The inflation and deflation cycles in the study consisted of 5 min intervals of boxing with BFR, followed by a one-minute rest period between game sets where BFR was not applied. After releasing the screws of the cuffs and manually deflating them to 0 mmHg, the cuffs were kept open for 60 s before being re-inflated. Additionally, the rate of perceived exertion (RPE) was recorded at 0, 5, 10, 15, and 20 min intervals during the exergame under both conditions using the 20-point Borg scale. HR was measured utilizing a cardiac Holter monitoring system, which is comprehensively described in the subsequent section. The rate pressure product (RPP) was computed as the product of HR and SBP [11], and mean arterial pressure (MAP) was calculated using the formula MAP = DBP + 1/3(SBP − DBP) [24].

Blood samples were collected from participants before and immediately after the boxing exergame for analysis of serum glucose, blood lactate concentration, normetanephrine, and GH levels. Post-exercise BP measurements were conducted under identical conditions to those of resting BP.

### 2.3. Measurement of HRV

The cardiac myPatch Holter monitoring system (Cardiac Monitoring Service, Newport Beach, CA, USA) was used to measure HRV [25]. This system facilitated the acquisition of data pivotal for the calculation of both frequency and time domain metrics of HRV in participants before and after exergame exercise sessions under BFR and normal conditions. In the frequency domain (which involves decomposing the heart rate signal into its frequency components), parameters such as high-frequency (HF) amplitude (0.15–0.40 Hz/ms^2^), very-low-frequency (VLF) waves, and total power (TP), which denotes the variance of all normal cardiac interbeat intervals (expressed in ms^2^), were computed. Concurrently, time domain parameters (based on the analysis of R-R intervals) were also analyzed, including the standard deviation of the average NN intervals (SDANN, which reflects the average standard deviation of successive R-R intervals, measured in milliseconds), the standard deviation of NN intervals (SDNN), indicative of the variability in intervals between consecutive normal heartbeats (in milliseconds), and NN50, representing the count of pairings of adjacent NN intervals differing by more than 50 milliseconds [26]. Adherence to a standardized protocol for HRV data collection was ensured for all participants. This involved pre-exercise preparation where a Holter device, equipped with four leads, was affixed to the participants’ torsos utilizing f-55 SKINTACT electrodes accompanied by poly-gel ultrasound gel. The leads were connected according to the standard operating procedures prescribed by the Holter system manufacturer, ensuring consistent and reliable data acquisition across participants.

### 2.4. Sampling of Glucose, Lactate, Growth Hormone, and Serum Normetanephrine

Fasted venous blood sampling was obtained from participants before each boxing exergame session, both under normal and BFR conditions. Prior to the commencement of the exercise protocol, blood samples were collected from the participants. Subsequently, the BFR cuffs were applied and inflated for those who were assigned to the BFR conditions. A second sample was obtained from participants immediately after the exergame concluded under normal and BFR conditions. Blood samples were immediately evaluated for all parameters of the study once obtained from participants according to the manufacturer’s procedures. More specifically, all blood draws were taken by a specialist (e.g., phlebotomist) from the brachial vein with a needle from the inner crease of the elbow. Lactate measurement was performed immediately after sampling utilizing the Enzyme Color Test method and GRAINER lactate analyzer (Greiner Bio-One International GmbH, Bio-one, Frickenhausen, Germany). Normetanephrine samples were transferred to a pre-cooled tube containing EDTA as an anticoagulant. The plasma was separated within 30 min by a refrigerated centrifuge and placed in plastic vials inside the freezer. Each sample was then analyzed by liquid chromatography–mass spectrometry (LC-MS/MS). Plasma glucose concentration was measured using the enzymatic colorimetric method (glucose oxidase, Pars Azmoun Company, Tehran, Iran) and Selectray 2 autoanalyzer. Growth hormone levels were measured using a LIAISON^®^ hGH kit (Diasorin, Saluggia, Italy) employing chemiluminescence technology (CLIA). All sampling and measurements were performed under controlled laboratory conditions and environments.

### 2.5. Statistical Analyses

The sample size was calculated using G*Power software (version 3.1.9.2) [27], employing an F-test for repeated measures, within–between interaction ANOVA. This analysis indicated that a total of 14 participants would be required to detect a medium effect size (Cohen’s f = 0.25) with a significance level (α) of 0.05 and a power of 80% for observing changes in HR and RPE following an acute exergaming session in young individuals [28]. The Shapiro–Wilk test was used to evaluate the normality of data distribution. The effects of training with and without BFR conditions on hemodynamic, metabolic, and hormone data were analyzed using a two × two analysis of variance (ANOVA) with repeated measures (time [pre-test vs. post-test] × group [with BFR vs. without BFR]) to determine the differences between the treatments over time. Also, to analyze RPE data, a five × two ANOVA with repeated measures (time [0 min vs. 5 min vs. 10 min vs. 15 min vs. 20 min] × group [with BFR vs. without BFR]) was used. Sidak’s multiple comparison test was used to detect between-group differences. The significance level was considered as *p* ≤ 0.05 for all statistical analyses. Data from cardiac Holter monitoring were used to determine the frequency range of the frequency domain parameters as well as the time domain parameters of the HRV, using computer analytic software associated with the myPatch Holter measurement system (Cardiac Monitoring Service, Newport Beach, CA, USA). All analyses and figure production were performed using Prism software (GraphPad Inc., San Diego, CA, USA, Release 8.4.3, 2020).

## 3. Results

The baseline characteristics of the participants are demonstrated in Table 1.

### 3.1. Hemodynamic and HRV Parameters

**Figure 1:**
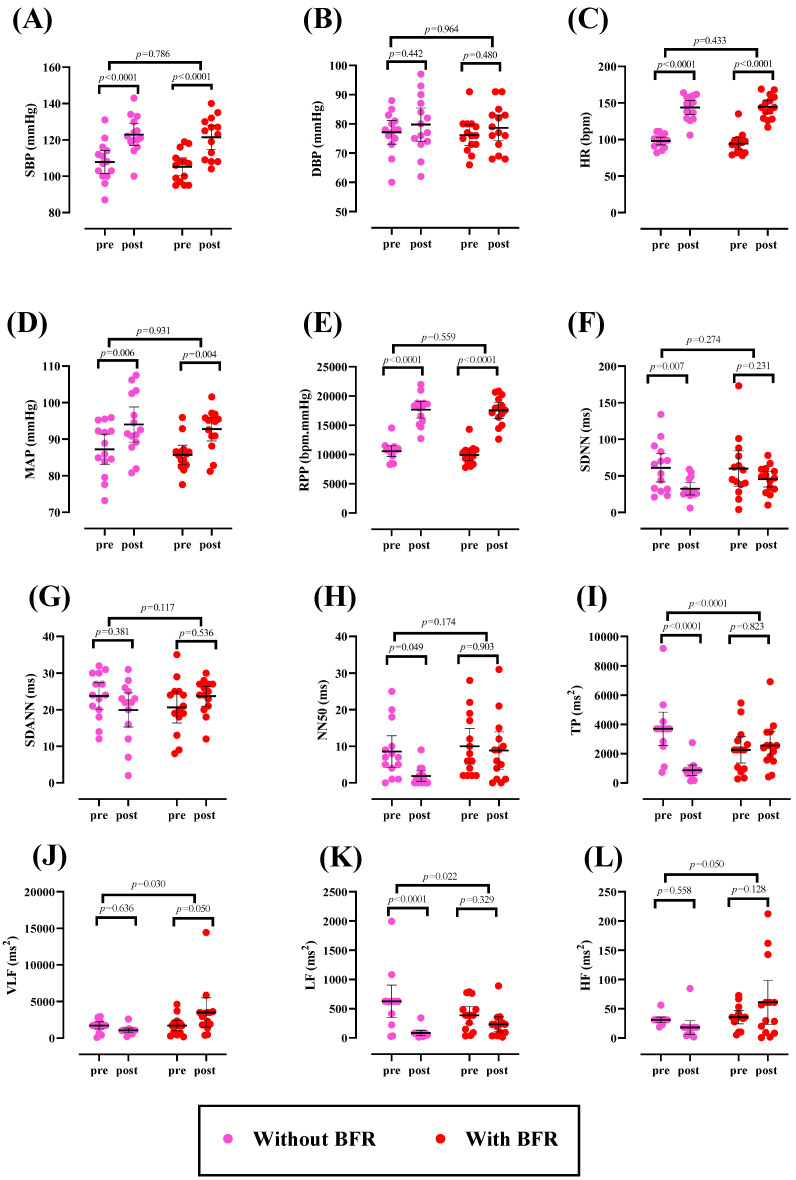
Changes in hemodynamic and HRV. (**A**) Systolic blood pressure (SBP); (**B**) diastolic blood pressure (DBP); (**C**) heart rate (HR); (**D**) mean arterial pressure (MAP); (**E**) rate pressure product (RPP); (**F**) standard deviation of normal-to-normal RR intervals (SDNN); (**G**) mean standard deviation of R wave intervals compared with the next R wave in milliseconds (SDANN); (**H**) number of successful RR intervals that are more than 50 milliseconds different (NN50); (**I**) total power or variance of all normal heart rate intervals in ms^2^ (TP); (**J**) very-low-frequency (VLF) waves; (**K**) low-frequency (LF) waves; and (**L**) amplitude of high-frequency waves at a range of 0.15–0.40 Hz/ms^2^ (HF). Error bars represent a 95% confidence interval (CI), and *p*-values above time points and between groups indicate time and time × group interaction, respectively.

There was no significant difference at baseline for any variable (*p* > 0.05). Hemodynamic and HRV data are shown in Figure 1. There was a significant time effect for SBP (*p* < 0.0001), HR (*p* < 0.0001), MAP (*p* < 0.0001), RPP (*p* < 0.0001), and SDNN (*p* < 0.0001). SBP [(without BFR: 15.14 mmHg; 95% CI: 8.585 to 21.70 mmHg); (with BFR: 16.21 mmHg; 95% CI: 9.65 to 22.77 mmHg)], HR [(without BFR: 45.79 bpm; 95% CI: 36.45 to 55.12 bpm); (with BFR: 50.21 bpm; 95% CI: 40.88 to 59.55 bpm)], MAP [(without BFR: 6.768 mmHg; 95% CI: 1.79 to 11.74 mmHg); (with BFR: 7.026 mmHg; 95% CI: 2.04 to 12 mmHg)], and RPP [(without BFR: 7086 bpm.mmHg; 95% CI: 5559 to 8613); (with BFR: 7625 bpm.mmHg; 95% CI: 6098 to 9151)] significantly increased, while SDNN [(without BFR: −28.57 ms; 95% CI: −49.95 to −7.19)] significantly declined from pre to post. However, DBP, SDANN, NN50, and HF remained unchanged over time (*p* > 0.05). Moreover, a significant time × group interaction was observed for TP (*p* = 0.0003), VLF (*p* = 0.0306), and LF (*p* = 0.0277). Significant reductions in TP and LF were identified in the condition without BFR (*p* < 0.0001) compared with the BFR group. Conversely, a significant increase in VLF was noted for the BFR group (*p* = 0.050) compared with the non-BFR group.

### 3.2. Metabolic Parameters

**Figure 2:**
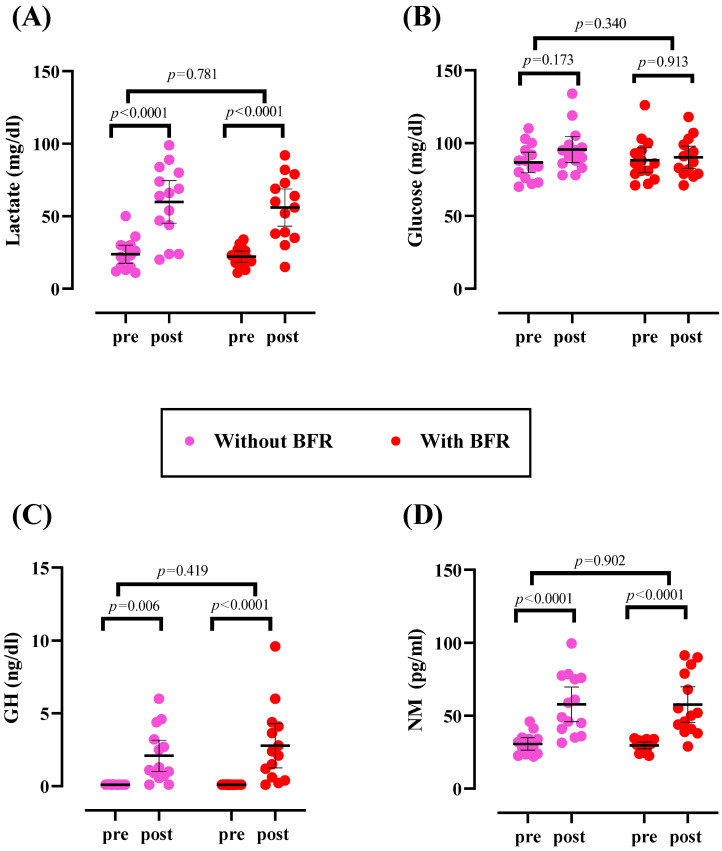
Changes in metabolic parameters and hormones. (**A**) Lactate; (**B**) glucose; (**C**) growth hormone (GH); and (**D**) normetanephrine (NM). Error bars represent a 95% confidence interval (CI), and *p*-values above time points and between groups indicate time and time × group interaction, respectively.

There was no significant difference at baseline for any variable (*p* > 0.05). Metabolic parameters and hormones are shown in Figure 2. There was a significant time effect for lactate (*p* < 0.0001). Lactate [(without BFR: 36.07 mg/dL; 95% CI: 22.80 to 49.35); (with BFR: 33.86 mg/dL; 95% CI: 20.58 to 47.13)] significantly increased from pre to post (*p* < 0.0001). However, blood glucose levels remained unchanged over time (*p* > 0.05).

### 3.3. Hormones

There was no significant difference at baseline for any variable (*p* > 0.05). There was a significant time effect for GH (*p* < 0.0001) and NM (*p* < 0.0001). GH [(without BFR: 1.97 ng/dL; 95% CI: 0.52 to 3.42); (with BFR: 2.68 ng/dL; 95% CI: 1.23 to 4.13)] and NM [(without BFR: 27.19 pg/mL; 95% CI: 14.62 to 39.75); (with BFR: −28.11 pg/mL; 95% CI: 15.55 to 40.68)] significantly increased from pre to post in both groups, with no significant differences between groups (*p* > 0.05).

### 3.4. RPE

**Figure 3:**
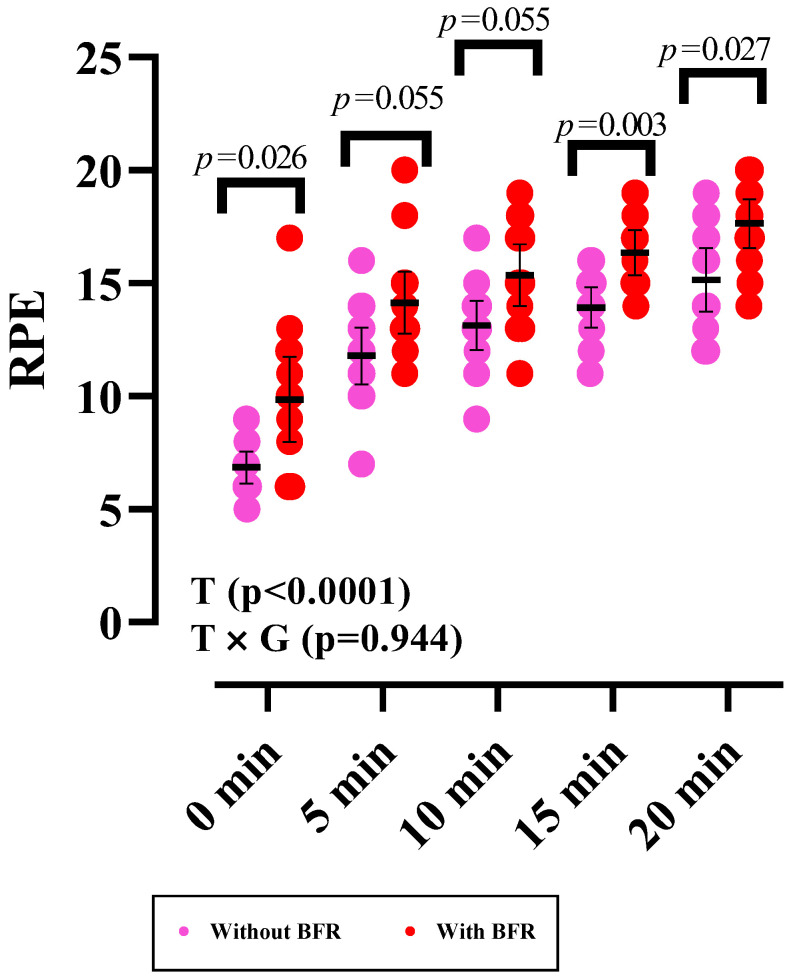
Changes in RPE values. Error bars represent a 95% confidence interval (CI), and *p*-values above time points and between groups indicate time and time × group interaction, respectively. T, time; T × G, time × group.

Changes in RPE are shown in Figure 3. All data for 0 min, 5 min, 10 min, 15 min, and 20 min revealed only a significant time effect for RPE (*p* < 0.0001). However, when assessing the between-group differences at each time point, there was a significant difference, with the BFR condition being greater at 0 min (mean difference: 3; 95% CI: 0.28 to 5.71; *p* = 0.026), 15 min (mean difference: 2.42; 95% CI: 0.70 to 4.15; *p* = 0.003), and 20 min (mean difference: 2.5; 95% CI: 0.21 to 4.78; *p* = 0.027).

## 4. Discussion

This study aimed to assess acute hemodynamic, metabolic, hormonal, HRV, and RPE responses to a boxing exergame session in non-athlete young individuals, comparing effects with and without BFR application. Findings indicate significant increases in VLF and RPE with the inclusion of BFR in a boxing exergame, whereas TP and LF diminished in the non-BFR condition. Hemodynamic, hormonal, and metabolic responses remained similar across conditions. This research is the first exploration of physiological responses to a boxing exergame with BFR application.

Prior investigations have illustrated that exergame interventions can improve the level of physical activity and increase HR and energy consumption among other acute responses in healthy and other populations such as adolescents/young adults [29,30]. As reported in our results, the level of serum normetanephrine significantly increased. Since epinephrine is metabolized to metanephrine and norepinephrine to normetanephrine, these free-circulating metanephrines (as catecholamine metabolites) are frequently used to detect increased sympathoadrenal function [31]. The significant increase in normetanephrine concentration following boxing exergame protocols may help to explain the increase in hemodynamic factors observed in this study [32]. Further, intensity-dependent alterations in cardiac autonomic activity facilitate increased HR, cardiac contractility, stroke volume, and cardiac output. Evidence suggests that intensity- and duration-dependent increases in muscle sympathetic nerve activity (SNA) to active and passive limbs during exercise are associated with increased release of norepinephrine from cardiac, renal, and visceral vessels [33]. While there were significant increases in hemodynamic variables for both groups, BFR in the present study was unable to elicit enhanced acute hemodynamic responses to boxing exergame exercise. However, there is evidence to suggest that aerobic exercise combined with BFR increases hemodynamic responses as well as energy expenditure during exercise compared with aerobic exercise sessions under normal (non-BFR) conditions [7]. Similarly, HR is understandably higher during high-intensity than low-intensity exercise, regardless of BFR application. BFR during low-intensity strength training (anaerobic) exercise appears to elicit similar responses as high-intensity exercise [34]. Evidence also suggests that aerobic exercise with BFR results in greater increased hemodynamic variables and higher energy demand during exercise compared with aerobic exercise sessions under normal conditions [31]. Altering the intensity, duration, or frequency of the boxing exergame may have elicited results similar to those mentioned above, warranting further investigation into BFR effects across different exercise intensities. Implementing a strategy where individuals are organized into pairs to participate in combat in a two-by-two format may foster enthusiasm among participants. This enhanced engagement is likely to amplify both psychological and physiological responses within each group, thereby reducing disparities in observed responses between the groups.

Acute and transient types of HRV have recently come under investigation during various conditions during physical activity, including BFR. According to research, a higher HR may lead to delayed HR adaptation and HRV in the transient kinetics of low-load exercise with BFR relative to exercise without BFR [35]. However, insignificant hemodynamic increases following BFR in the present study may partially be explained by the level of restriction applied during exercise. Such differences observed between studies appear to be influenced by the intensity and duration of the implemented exercise protocols, as well as differences in the type of equipment used for BFR. Thus, there is little homogeneity for BFR protocols at present in the literature.

In the present study, both protocols, with and without BFR, decreased the SDNN parameter. However, this decrease was only significant in the group without BFR. Also, other time-based HRV parameters, such as SDANN and NN50, indicated a decreasing trend in the normal group; this result is significant in the case of NN50, which appears consistent with data presented in previous studies [36,37]. As a time-based indicator, SDNN elucidates the total variability and overall autonomic nervous system activity in HRV [38]. The immediate withdrawal of parasympathetic flow during the onset of exercise gives way to increased sympathetic activity, causes upregulation of the heart and blood vessels, and results in decreased HRV seen when moving from rest to exercise. Such a reduction in HRV will continue for the duration of exercise [39]. This reduced SDNN response may, in part, be explained by the significant elevation of normetanephrine concentration following the boxing exergame protocol. Despite the increase in normetanephrine, there was no significant difference between groups in the current study. This could be partly due to the statistical power to identify such changes or that simply the BFR protocol was too conservative.

In the protocol without BFR, the HRV frequency parameters (HF, VLF, and TP) decreased, albeit insignificantly in the case of HF, which is consistent with prior research that reported a significant decrease in VLF during treadmill running [35]. Frequency-based HRV parameters, such as HF and LF, reflect the neural activity of regulatory mechanisms during exercise. Time-based parameters of HRV, along with parameters of high-frequency waves, are used clinically in determining vagal (parasympathetic) tone. It has been widely believed that HF power primarily reflects the parasympathetic cardiac drive and that LF power has a predominantly sympathetic component [40]. Further, significant reductions in the TP parameter observed in the present study under normal conditions also suggest reduced HRV and activity of the cardiac autonomic system [41]. The fact that these same observations were not observed under BFR in our study indicates improved HRV outcomes following exercise during this condition. Further, increased SDANN, TP, and HF (insignificant) observed in the present study under the BFR condition may have been associated with cardiac parasympathetic activity (e.g., HF) seen during this condition. Within the BFR group, we observed a notable upsurge in the VLF component. The VLF component of HRV is believed to be influenced by various factors including the sympathetic nervous system, parasympathetic nervous system, and non-autonomic factors. An increase in VLF post-exercise might suggest changes in these systems’ activities due to the stress of exercise. This result is consistent with previous research, which identified an increase in VLF power during rhythmic activity (an alternating pattern of rest and mild exercise) in contrast to periods of rest [42]. It is important to note that HRV, which often augments when either vagal tone is elevated or when it supersedes sympathetic dominance [43], does not appear to be significantly impacted by BFR. The observed effects of blood flow restriction (BFR) on cardiovascular regulation during exercise may be linked to autonomic nervous system modulation. Specifically, BFR’s impact on HRV could be due to altered baroreceptor reflex function. This reflex, regulating BP via HR and vascular tone adjustments, receives autonomic signals relayed to the hypothalamus, affecting muscle blood flow and BP [44]. BFR might alter baroreceptor sensitivity in the carotid sinus and aortic arch, which detects BP changes [45]. Additionally, non-neuronal mechanisms, such as the mechanical effects of breathing on the SA node, might contribute to BFR’s effects [34]. Our study’s alternating BFR application may have prompted parasympathetic activation and sympathetic withdrawal, explaining HRV variations post-exercise under BFR and non-BFR conditions. Higher HRV in BFR participants could stem from increased training stimulus affecting perceived effort and leading to divergent HRV responses. BFR’s cumulative effect on HRV likely depends on exercise parameters and individual factors like age, health, fitness, and genetics. Further investigation is needed to elucidate these complex interactions and BFR’s role in HRV modulation.

Of the metabolic indices analyzed, a significant difference was observed between the amount of lactate before and after exergame in both conditions (with and without BFR). Lactate production, a byproduct of anaerobic ATP synthesis, increases and accumulates in blood via monocarboxylate transporters during intense exercise [46]. BFR, by impairing blood flow and muscle oxygenation, has been shown to elevate blood lactate concentrations [47]. Okita et al. found that low-intensity exercise without BFR does not significantly alter intracellular pH. However, intermittent and continuous BFR significantly decreased pH, correlating with increased lactate and altered acid/base balance [48]. Despite these findings, our study showed no significant lactate differences between groups. In contrast, Corvino et al. [49] and Thomas et al. [50] reported increased lactate levels with BFR during aerobic activities. Similarly, low-intensity cycling with BFR was found to elevate metabolic and cardiovascular stress compared with normal conditions [51]. However, Mirzaei et al. observed unchanged lactate concentrations following aerobic exercise with BFR [52]. The discrepancies among these studies may be attributed to varying BFR cuff pressures, exercise intensity and duration, and exergame type among participants. Moreover, psychological factors may also play a role in the discrepancies among studies. Recent research indicates that the production and accumulation of lactate in the body may be affected by factors such as mood, emotional state, pain tolerance, pain perception, concentration, as well as stress and anxiety [53,54,55].

There was an insignificant difference in the present study between serum glucose levels before and after the protocol under the condition without BFR. Interestingly, a slight yet insignificant increase in glucose levels was noted following the BFR condition compared with the baseline. Factors involved in stimulating glucose production during exercise remain under investigation and it has been hypothesized that during high-intensity exercise, control of glucose production shifts from pancreatic hormones (e.g., insulin) to catecholamines. Norepinephrine and epinephrine in blood circulation can increase by 10 to 20 times their resting values [56]. BFR training enhances oxidative stress and 5′-AMP-activated protein kinase (AMPK) signaling, regulating glucose uptake and glucose transporter (GLUT4) transport during and post-exercise. Tissues with restricted blood flow increase glucose uptake by relocating GLUT4 to the sarcolemma. BFR preconditioning also activates AMPK via protein kinase C (PKC), boosting GLUT4 expression [57]. We suggest that the lack of between-group differences for glucose levels is indicative of insufficient differences in exercise intensity of the boxing exergame under the BFR and normal conditions. Furthermore, based on the increased normetanephrine observed for both conditions (with and without BFR) in this research, a plausible explanation for the increased blood sugar response immediately post-exercise may be attributed to normetanephrine release. Regular measurements of glucose levels following exercise may better help to explain potential differences when BFR is applied, which is a limitation of the current research investigation.

Hormonal analyses revealed significant GH and normetanephrine differences pre- and post-exergame in both groups, though no between-group differences emerged for GH or normetanephrine, with BFR groups showing minor increases. Exercise enhances sympathetic nervous system activity, upregulating catecholamine release, and thus stimulating GH secretion [15]. Increases in GH levels after a low-intensity training session with BFR have been shown to elevate threefold over the baseline in a cohort of college-aged females [58]. Similarly, Ozaki et al. demonstrated that walking with BFR increased GH levels post-exercise five times above resting values in a group of young men [59]. Therefore, BFR seems to play a more significant role in facilitating increased serum GH levels than exercise alone. It has been suggested that greater stimulation of peripheral afferent nerves, especially peripheral nerves of fast-twitch muscle fibers used preferentially during higher-intensity exercise [60], may explain relatively higher GH after low-intensity aerobic exercise with BFR [59]. In this situation, BFR may result in the preferential recruitment of anaerobic muscle fibers, which rely less heavily on oxygen availability compared with the more aerobic slow-twitch fibers. In addition, activation of Erk 1.2 (not investigated in the present study) is sensitive to not only the number of contractions during exercise, but exercise intensity as well. Since Erk 1.2 phosphorylation rises as exercise intensity and the number of skeletal muscle contractions increases, exercise intensity or lack of sufficient BFR stimulus in the present study may explain the lack of between-group differences for GH. In this regard, studies have shown that aerobic exercise combined with BFR activates the phosphorylation of various proteins that are involved in both the mechanical target of rapamycin (mTOR) and mitogen-activated protein kinase (MPAK), which itself can stimulate the secretion of anabolic hormones such as GH [59]. While the difference in GH was not significant between groups, GH values trended higher post-exercise in the BFR group. One plausible explanation for the lack of significance for GH between groups may lie in its parallelism with the normal group and the amount of normetanephrine released after exergames in both protocols [61]. Playing a boxing exergame in the present study with BFR may not have stimulated sympathetic activation similar to other protocols utilized in prior investigations [56,58], resulting in less GH secretion. We suggest that the intensity, duration, type of exercise protocol, and even the level of arterial pressure under the BFR condition may explain the similar GH levels observed in pre- to post-boxing exergame results in both groups.

When comparing the RPE reported by participants, a significant difference was observed between the groups, with RPE being greater in the BFR condition at 0 min, 15 min, and 20 min of exercise. Upon transitioning from rest to exercise, skeletal muscle contractions are enhanced by parasympathetic inhibition and, subsequently, by mechanical muscle reflexes and gradual sympathetic activation, such as the release of catecholamines. Furthermore, low-load exercise with BFR is believed to increase muscle metabolites resulting from tissue hypoxia, inadequate removal from the limited venous return, and shifting metabolic and neuromuscular reflex state [35], which may contribute to exaggerated sensations of perceived exertion as observed in the present study.

This study has several limitations that should be acknowledged. Firstly, the relatively small sample size may constrain the generalizability of the findings to a broader population, as the diminutive sample may not adequately represent the larger community, thereby limiting the broader applicability of the study’s outcomes. Another limitation is the absence of gender as a factor in the analysis of the study’s results. Ignoring the potential influence of gender on the outcomes may overlook important differences in responses to BFR exergaming between males and females. The study’s emphasis on acute responses immediately following a single session of a boxing exergame restricts the generalizability of conclusions regarding the long-term effects of incorporating BFR into exergaming routines. The scope of applicability to diverse demographic profiles is hindered by the homogeneity of the participant sample, comprising exclusively young individuals with similar age distributions and BMIs. Moreover, considering the study’s aim to evaluate the additive stress of BFR in exergaming and the hypothesis that BFR induces significant physiological modifications, the inclusion of a placebo group becomes both pertinent and essential. Consequently, it is strongly recommended for future investigations to incorporate a placebo group. Additionally, the study’s emphasis on immediate outcome measures neglects the assessment of long-term effects, while the external validity of the results may be subject to scrutiny given the controlled laboratory environment. An additional limitation of our current research is the inability to examine certain research-related variables, especially muscle recruitment as assessed through electromyography, due to the lack of necessary research infrastructure. In consideration of these limitations, further inquiry is warranted to mitigate potential biases, evaluate the long-term implications, and explore the practical feasibility, thereby attaining a more comprehensive understanding of the effects of BFR exergaming.

## 5. Conclusions

This study investigated the effects of BFR during a boxing exergame session on non-athlete young individuals. The results showed that hemodynamic, hormonal, and metabolic responses were similar regardless of whether or not BFR was applied. However, there were differences in specific HRV markers and participants’ perceived exertion levels. BFR led to an increase in VLF and perceived exertion while also mitigating decreases in LF and TP observed without BFR during exergaming. These findings suggest that BFR may influence cardiac autonomic responses and enhance perceived effort during exergaming without significant effects on other physiological parameters. This study provides valuable insights into the design of exergame sessions and the use of BFR in young populations. Future research should focus on manipulating exercise parameters and exploring the optimal use of BFR in exergaming.

## 6. Future Recommendations

The findings of this investigation indicate the need for subsequent research to examine the enduring implications of incorporating BFR into exergaming regimens over an extended temporal duration. This inquiry would provide elucidation on the enduring benefits and potential drawbacks associated with BFR training. Additionally, investigating the impact of repeated exergaming sessions with BFR on muscle strength, endurance, and cardiovascular fitness would contribute to a more comprehensive understanding of the physiological modifications induced by this training modality. Undertaking comparative analyses that encompass diverse BFR levels and durations during exercise is imperative to ascertain the optimal restriction for eliciting desired physiological responses while ensuring safety. Further research is warranted to gain a comprehensive understanding of the physiological mechanisms underlying BFR’s influence on cardiac autonomic responses and perceptual exertion during exercise. Furthermore, exploring the effects of BFR exergaming across various populations, including older individuals and those with metabolic or cardiovascular conditions, would provide insights into its potential, effectiveness, and safety.

## Figures and Tables

**Table 1:** Baseline characteristics of the participants.

Variable	With BFR	Without BFR
	Measure	
	Anthropometry	
	Mean ± SD	
Age (y)	29.93 ± 7.04	
Body mass (kg)	63.30 ± 14.04	
Stature (cm)	171.30 ± 8.53	
BMI (kg.m^−2^)	22.40 ± 3.469	
	Hemodynamic and HRV	
SBP (mmHg)	105.2 ± 8.58	107.7 ± 11.0
DBP (mmHg)	76.14 ± 6.04	77.14 ± 7.01
HR (bpm)	92.00 ± 9.29	98 ± 9.11
MAP (mmHg)	85.73 ± 4.52	87.25 ± 7.06
RPP (bpm.mmHg)	9666.1 ± 1120	10,576 ± 1588.2
SDNN (ms)	59.8 ± 41.9	61 ± 33.7
SDANN (ms)	20.6 ± 7.65	23.7 ± 6.50
NN50 (ms)	7.27 ± 6.16	7.17 ± 6.30
TP (ms^2^)	2253.3 ± 1559.7	3149.34 ± 835.12
VLF (ms^2^)	1721.6 ± 1264.2	1717.3 ± 855.73
LF (ms^2^)	388.03 ± 259.02	500.17 ± 230.94
HF (ms^2^)	35.80 ± 24.9	31.1 ± 13.2
	Metabolic parameters	
Lactate (mg/dL)	22.14 ± 6.75	23.79 ± 10.85
Glucose (mg/dL)	88.29 ± 14.64	86.71 ± 12.24
	Hormones	
GH (ng/dL)	0.104 ± 0.01	0.107 ± 0.01
NM (pg/mL)	29.59 ± 4.02	30.68 ± 7.29
	RPE	
0 min	9.86 ± 3.27	6.86 ± 1.23
5 min	14.14 ± 2.38	11.79 ± 2.19
10 min	15.36 ± 2.37	13.14 ± 1.87
15 min	16.36 ± 1.73	13.93 ± 1.54
20 min	17.64 ± 1.86	15.14 ± 2.44

Data are reported as mean ± SD. Abbreviations: BMI, body mass index; SBP, systolic blood pressure; DBP, diastolic blood pressure; HR, heart rate; MAP, mean arterial pressure; RPP, rate pressure product; SDNN, standard deviation of the intervals of two regular beats in milliseconds; SDANN, mean standard deviation of R wave intervals compared with the next R wave in milliseconds; NN50, number of successful RR intervals that are more than 50 milliseconds different; TP, total power or variance of all normal heart rate intervals in ms^2^; VLF, very-low-frequency waves; LF, low-frequency waves; HF, amplitude of high-frequency waves at a range of 0.15–0.40 Hz/ms^2^; GH, growth hormone, NM, normetanephrine; and RPE, borg rating of perceived exertion.

## Data Availability

Data sharing is applicable.

## Abbreviations

AMPK	5′AMP-activated protein kinase	BFR	Blood flow restriction
BP	Blood pressure	CLIA	Chemiluminescence technology
DBP	Diastolic blood pressure	GLUT4	Glucose transporter
GH	Growth hormone	HF	High frequency
HR	Heart rate	HRV	Heart rate variability
LF	Low frequency	LC-MS/MS	Liquid chromatography–mass spectrometry
MAP	Mean arterial pressure	MPAK	Mitogen-activated protein kinase
mTOR	Mechanical target of rapamycin	NM	Normetanephrine
NN50	Number of pairs of adjacent NN intervals differing by more than 50 ms	PAR-Q PKC	Physical Activity Readiness Questionnaire Protein kinase C
RPE	Rating of perceived exertion	RPP	Rate pressure product
SBP	Systolic blood pressure	SDANN	Standard deviation of the average NN intervals
SDNN	Standard deviation of NN intervals	SNA	Sympathetic nerve activity
TP	Total power	VLF	Very low frequency
